# The microRNA *miR‐134‐5p* induces calcium deposition by inhibiting histone deacetylase 5 in vascular smooth muscle cells

**DOI:** 10.1111/jcmm.15670

**Published:** 2020-08-11

**Authors:** Nakwon Choe, Sera Shin, Hosouk Joung, Juhee Ryu, Young‐Kook Kim, Youngkeun Ahn, Hyun Kook, Duk‐Hwa Kwon

**Affiliations:** ^1^ Department of Pharmacology Chonnam National University Medical School Hwasun Republic of Korea; ^2^ Department of Biochemistry Chonnam National University Medical School Hwasun Republic of Korea; ^3^ Department of Cardiology Chonnam National University Hospital Gwangju Republic of Korea

**Keywords:** histone deacetylase 5, microRNA, *miR‐134‐5p*, vascular calcification, vascular smooth muscle cells

## Abstract

Calcium deposition in vascular smooth muscle cells (VSMCs) is a form of ectopic ossification in blood vessels. It can result in rigidity of the vasculature and an increase in cardiac events. Here, we report that the microRNA *miR‐134‐5p* potentiates inorganic phosphate (Pi)‐induced calcium deposition in VSMCs by inhibiting histone deacetylase 5 (HDAC5). Using miRNA microarray analysis of Pi‐treated rat VSMCs, we first selected *miR‐134‐5p* for further evaluation. Quantitative RT‐PCR confirmed that *miR‐134‐5p* was increased in Pi‐treated A10 cells, a rat VSMC line. Transfection of *miR‐134‐5p* mimic potentiated the Pi‐induced increase in calcium contents. *miR‐134‐5p* increased the amounts of bone runt‐related transcription factor 2 (RUNX2) protein and bone morphogenic protein 2 (BMP2) mRNA in the presence of Pi but decreased the expression of osteoprotegerin (OPG). Bioinformatic analysis showed that the HDAC5 3′untranslated region (3′UTR) was one of the targets of *miR‐134‐5p*. The luciferase construct containing the 3′UTR of HDAC5 was down‐regulated by *miR‐134‐5p* mimic in a dose‐dependent manner in VSMCs. Overexpression of HDAC5 mitigated the calcium deposition induced by *miR‐134‐5p*. Our results suggest that a Pi‐induced increase of *miR‐134‐5p* may cause vascular calcification through repression of HDAC5.

## 1 INTRODUCTION

Certain chronic stresses and other metabolic or cardiovascular diseases may cause an abnormal deposition of calcium phosphate crystals in blood vessels. Those stresses can be initiated by atherosclerosis, chronic kidney disease and diabetes. This ectopic ossification induces remodelling and rigidity of the blood vessels, which causes ischaemia and impairment of circulation.^1^ By examining the anatomy of the blood vessel, we can distinguish whether the vascular calcification is intimal calcification or medial calcification. Intimal calcification is often associated with atherosclerosis, whereas medial calcification is related to metabolic diseases such as diabetes mellitus or chronic kidney disease.^2^ Although the main causative diseases may differ, no clear pathophysiologic differences within the vascular smooth muscle cells (VSMCs) have been described, which suggests that the extracellular stimuli share a common final intracellular signal pathway or pathways during calcification.

Noncoding RNA represents DNA sequences that are transcribed but not translated into proteins. Although their roles are still being investigated, recent advances show that noncoding RNAs have a unique function to regulate pathophysiologic events as well as cellular homeostasis.^3^ Noncoding RNA can be classified into groups by differences in length, structure and function. Usually, sequences spanning less than 200 nucleotides are named small noncoding RNAs, and microRNAs (miRNAs) are one of the small noncoding RNAs. miRNAs commonly act as negative regulators of other coding genes by inducing degradation of target gene mRNA. They bind to the 3′UTR of the target mRNA, resulting in either degradation of mRNA or inhibition of translation.^4^ It is noteworthy that a perfect match between the miRNA and the target 3′UTR is not needed and that a single coding gene can provide multiple sites for many miRNAs.^5^ This broad specificity results in diverse and complex regulation of cellular functions by miRNAs. Indeed, it has been reported that over 90% of human genes are under the control of miRNAs.^6^

Likewise, many functions of VSMCs have been reported to be regulated by miRNAs. Indeed, atherosclerosis and VSMC proliferation provide good examples of miRNA‐mediated regulation.^7^ We found that *miR‐132*, *miR‐34c* and *miR‐124* modulate VSMC proliferation and thereby affect atherogenesis.^8^, ^9^, ^10^ Vascular calcification is another disease that is tightly regulated by miRNA. Indeed, miRNAs may function as negative regulators in vascular calcification^11^, ^12^ or as positive initiators.^13^, ^14^ Considering the complexity of miRNA‐mediated regulation of diseases, however, further investigation is needed to understand the development of vascular calcification.

HDAC5, a member of the Class II histone deacetylases (HDACs), plays a role in cellular and epigenetic processes that control the progression of diverse diseases, including cancer,^15^ cardiac diseases^16^ and vascular calcification.^14^ Several studies have reported that miRNAs such as *miR‐124/miR‐9*, *miR‐2861* and *miR‐589* target HDAC5 in neurons,^17^ bone^18^ and lung.^19^ For example, Xia et al reported that *miR‐2861* directly targets HDAC5 and promotes osteogenic transdifferentiation of VSMCs by inhibiting the expression of RUNX2.^14^ In the present study, we investigated the role of *miR‐134‐5p* in calcium deposition in VSMCs. We found that HDAC5 expression is down‐regulated by *miR‐134‐5p*, thereby increasing calcium deposition in VSMCs. These findings give novel insight into a role of *miR‐134‐5p* as a regulator of HDAC5 in calcium deposition in VSMCs.

## 2 MATERIALS AND METHODS

All experimental procedures were approved by the Chonnam National University Medical School Research Institutional Animal Care and Use Committee and followed the National Institutes of Health *Guide for the Care and Use of Laboratory Animals* (NIH Publication No. 8023, revised 1978).

### 2.1 miRNA mimic, small interfering RNA (siRNA) and antibodies

*miR‐134‐5p* mimic, control miRNA, *HDAC5* siRNA and scramble siRNA were purchased from Bioneer Corp. Antibodies against RUNX2 (23981, Abcam) and glyceraldehyde 3‐phosphate dehydrogenase (GAPDH) (G9545, Sigma) were used at 1:1000 dilution.

### 2.2 Cell cultures

Rat VSMCs were isolated from thoracic aorta of 6‐7‐week‐old male Sprague‐Dawley rats after anaesthesia with 2,2,2‐Tribromoethanol (240 mg/kg; intraperitoneal injection) (T48402, Sigma). The aorta was washed using ice‐cold phosphate‐buffered saline (PBS) before incubation in Ham's F12 medium (12‐615F, Lonza) with 0.2% collagenase I (LS004196, Worthington) at 37°C for 30 minutes. The aorta was opened longitudinally, and the intima was scraped from the luminal surface. Tissue samples were minced in Ham's F12 media containing 300 U/mL penicillin and 300 U/mL streptomycin and then incubated in 0.2% collagenase I solution at 37°C for 30 minutes. The rat VSMCs were cultured in DMEM (LM001‐05, Welgene) with 10% foetal bovine serum (FBS) (S001‐07, Welgene) and antibiotics (15240062, ThermoFisher Scientific). Rat VSMCs were used at passages 2‐6.

A10 cells, derived from embryonic rat aorta, were purchased from American Type Culture Collection (CRL‐1476) and have been used as a model system of rat VSMCs. The A10 cells were cultured in DMEM with 10% FBS. All cells were maintained in an incubator under a humidified atmosphere with 5% CO_2_ at 37°C.

### 2.3 Induction of vascular calcification in vitro

The cell culture medium supplemented with 2 mmol/L Pi was changed every 2 days for up to 6 days to induce calcification. The cells were washed twice with PBS before quantification of calcium deposition.

### 2.4 miRNA and mRNA microarray and bioinformatics

To investigate changes in levels of miRNA in Pi‐treated rat VSMCs, miRNA microarray was performed after pooling of three samples. RNA was isolated as described below. To reduce the experimental error, two pooled samples were independently used for the microarray and the averaged values were evaluated further.

The mRNA samples were analysed by utilizing the mRNA microarray (Agilent Microarray, Agilent‐028282), and the results were previously deposited in Gene Expression Omnibus (GEO) database under accession code GSE74755.^20^ Putative target mRNAs of *miR‐134‐5p* were screened using Targetscan (http://www.targetscan.org/),^21^ microRNA.org (http://www.microrna.org) ^22^ and mirDB (http://mirdb.org/).^23^

For the clustering analysis of miRNA microarray data, Cluster 3.0 was used for the unsupervised hierarchical clustering.^24^ The analysis result was visualized by Java Treeview.^25^ In the clustering analysis, microarray signals were median‐centred and normalized for genes and arrays. Average linkage analysis was performed using the centred‐correlation method.

### 2.5 Quantification of calcium deposition

Cells were decalcified in 0.6 N HCl at 4°C for 24 hours. The calcium content of the HCl supernatants was determined using QuantiChrom^TM^ Calcium Assay Kit (DICA‐500, BioAssay Systems) according to the manufacturer's protocol. Briefly, the samples had been mixed with working reagent, and then the absorbance of the mixture at 570 nm was measured using ELx808 Absorbance Reader (BTELX808, BioTek Instruments). Decalcified cells were lysed with 0.1 N NaOH/0.1% SDS to extract proteins. The protein content was quantified with BCA Protein Assay kit (23225, ThermoFisher Scientific). The calcium content was normalized against the protein content.

### 2.6 Reverse transcription and quantitative real‐time polymerase chain reaction (qRT‐PCR)

Total RNA was extracted using either TRIzol Reagent (15596026, Invitrogen) or NucleoSpin^®^ RNA/Protein (740933.250, Macherey‐Nagel) following the manufacturer's protocols. mRNAs were reverse‐transcribed using the SuperScript™ First‐Strand Synthesis System and random hexamer primer (11904018, SO142, ThermoFisher Scientific). The cDNAs were then analysed by qPCR using a QuantiTect SYBR Green PCR Kit (204141 and Qiagen), gene‐specific primers and a Rotor gene Q real‐time PCR cycler (9001550, Qiagen). *18S rRNA* was used as an expression control. Pre‐designed qPCR primer for 18S rRNA was purchased from ThermoFisher Scientific (Rn03928990_g1). Custom‐designed primers for HDAC5 (sense 5′‐TCC CGT CCG TCT GTC TGT TA‐3′, antisense 5′‐GAC ATG CCA TCC GAC TCG TT‐3′), BMP2 (sense:5′‐TCA CCC CGG CTG TGA TGC GA‐3′, antisense: 5′‐ACC CGC AAC CCT CCA CAA CC‐3′) and OPG (sense: 5′‐GGC AGG GCA TAC TTC CTG TTG CC‐3′, antisense: 5′‐TCG GTT GTG GGT GCG GTT GC‐3′) were purchased from Bioneer (Daejon, Korea).

The cDNA of *miR‐134‐5p* was synthesized by adding a poly (A) tail to the 3′ end and ligating an ‘adapter’ to the 5′ end of an miRNA followed by reverse transcription with universal primer using the TaqMan Advanced miRNA cDNA Synthesis Kit (A28007, Applied Biosystems). The cDNAs were then analysed by qPCR using a QuantiTect SYBR Green PCR Kit (204141, Qiagen, Hilden, Germany) with gene‐specific primers for *miR‐134‐5p* and *18S rRNA* (4427975 and 4333760F, Applied Biosystems).

### 2.7 Western blot analysis

Cellular proteins were prepared with lysis buffer [50 mmol/L Tris (pH 8.0), 150 mmol/L NaCl, 1 mmol/L EDTA, 1% NP‐40 (28324, ThermoFisher Scientific), 1 mmol/L dithiothreitol (DTT), 1 mmol/L phenylmethylsulfonyl fluoride, 1 mmol/L Na_3_PO_4_ and protease inhibitor (11 697 498 001, Hoffmann‐La Roche [Basel, Switzerland])]. The proteins were separated by sodium dodecyl sulphate‐polyacrylamide gel electrophoresis (SDS‐PAGE) and then transferred to a polyvinylidene difluoride membrane (Millipore), followed by blocking with 5% skim milk (232100, BD Difco) in TRIS‐buffered saline‐Tween 20 (20605, ThermoScientific Fisher) (TBST). The membranes were then incubated with primary antibodies overnight at 4°C on a rocker. After three washes in TBST, the membranes were incubated with horseradish peroxidase‐conjugated secondary antibodies (7076, 7074, Cell Signaling) for 1 hour at room temperature. The peroxidase activity was visualized by enhanced chemiluminescence using Western Blotting Luminol Reagent (sc‐2048 Santa Cruz Biotechnology) and FUJIFILM Luminescent Image Analyzer LAS‐3000 (Fujifilm Life Science). Quantification of Western blot analysis was performed after retrieving the density of the bands using Scion Image software (Scion Corporation) after more than three independent sets of experiments.

### 2.8 Cloning

The coding sequence of rat *HDAC5* was cloned onto *pcDNA6/myc‐His vector* (V22120, ThermoFisher Scientific) for overexpression of HDAC5 in mammalian cells. A DNA fragment corresponding to the 3′UTR of rat *HDAC5* containing the putative binding site for *miR‐134‐5p* was cloned into *psiCHECK™‐2* vector (C8021A, Promega) for the luciferase assay.

### 2.9 Luciferase assay

The Renilla luciferase vector, with firefly luciferase as an internal control, was co‐transfected with *miR134‐5p* mimic into A10 cells, and luciferase activity was measured by using the Luciferase Assay System (E1500, Promega) following the manufacturer's protocols. Renilla luciferase activity was normalized against firefly luciferase activity.

### 2.10 Statistical analysis

Data are presented as mean ± SEM Statistical significance was determined by Student's *t* tests or one‐way ANOVA, followed by Tukey's honestly significant difference multiple‐comparison post hoc test using PASW Statistics 19 software (SPSS, an IBM Company).

## 3 RESULTS

### 3.1 Screening of vascular calcification‐associated miRNAs and their possible targets

**FIGURE 1:**
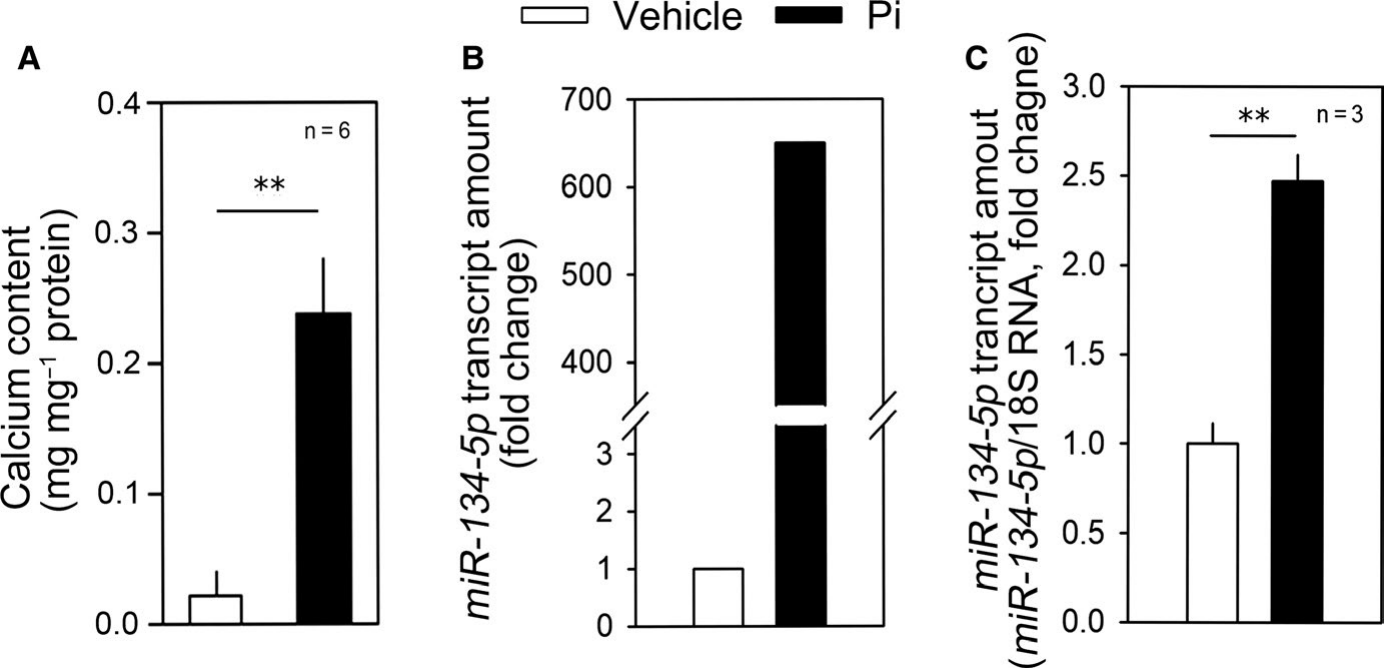
*miR‐134‐p5* is up‐regulated by treatment with inorganic phosphate. A, Treatment with inorganic phosphate (Pi) significantly induced calcium deposition in rat vascular smooth muscle cells (RVSMCs). B, microRNA microarray analysis showing the increase in *miR‐134‐5p* in response to Pi in rat VSMCs. Two values were averaged. C, Treatment with Pi for 6 d significantly increased the *miR‐134‐5p* transcript amount in A10 cells. Pi, inorganic phosphate. **P < .01

We used microRNA array analysis to look for miRNAs associated with vascular calcification. We first treated rat VSMCs with Pi to induce sufficient calcium deposition (Figure 1A). We next performed miRNA microarray (GSE 130 486). Among eight significantly altered miRNAs, we noticed that *miR‐134‐5p* was increased over 600‐fold (Figure 1B). The up‐regulation of *miR‐134‐5p* was further confirmed by qRT‐PCR (Figure 1C). We were interested in *miR‐134‐5p* because it has been reported that it is one of the miRNAs up‐regulated in the serum of patients with coronary artery calcification.^26^ Also, in endothelial cells, *miR‐134‐5p* is related to tumour angiogenesis^27^ and endothelium‐associated cardiac tube formation during development.^28^ More recently, *miR‐134‐5*p was shown to be involved in VSMC phenotypic switching and migration and in the progression of thoracic aortic dissection.^29^ However, its role in calcium deposition in VSMCs has not been reported.

### 3.2 *miR‐134‐5p* induces calcium deposition

**FIGURE 2:**
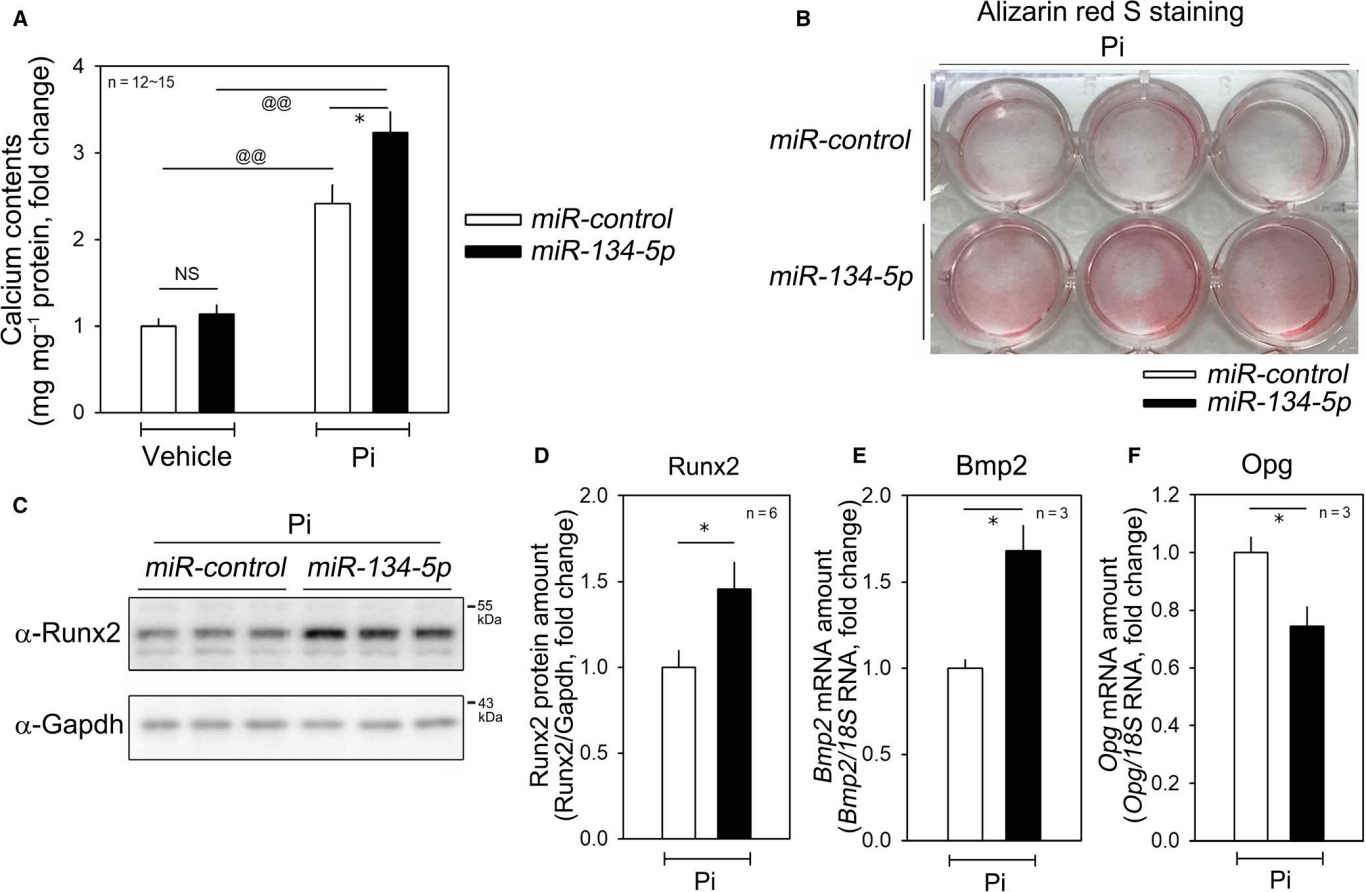
*miR‐134‐5p* potentiates vascular calcification. A, In the absence of Pi, transfection of *miR‐134‐5p* mimic did not significantly affect calcium contents in A10 cells. However, in the presence of Pi, *miR‐134‐5p* enhanced calcium deposition. B, Alizarin red S staining in Pi‐treated A10 cells. C‐D, Transfection of *miR‐134‐5p* mimic increased the protein expression of RUNX2. C, Representative Western blot image. D, Quantitative results. E, Transfection of *miR‐134‐5p* mimic increased the mRNA amount of bone morphogenic protein 2 (BMP2) in the presence of Pi in A10 cells. F, *miR‐134‐5p* decreased the mRNA amount of osteoprotegerin (OPG). * *P* < .05, ^@@^ *P* < .01. NS, not significant

We next investigated the role of *miR‐134‐5p* in calcium deposition in VSMCs. Transfection of *miR‐134‐5p* mimic did not increase calcium deposition (left two bars in Figure 2A). However, it potentiated Pi‐induced vascular calcification. Treatment with Pi for 6 days induced calcium deposition, which was further potentiated by transfection of *miR‐134‐5p* mimic (right two bars in Figure 2A). Alizarin red S staining showed that Pi‐induced calcium deposition was enhanced by transfection of *miR‐134‐5p* mimic (Figure 2B).

Vascular calcification is a form of ectopic bone formation and shares a common pathway with osteogenic signals in bone.^30^ Among the bone‐forming signals, RUNX2 is a key player in osteoblast differentiation.^31^ As in bone formation, RUNX2 is highly associated with vascular calcification.^32^ Interestingly, *miR‐134‐5p* increased the RUNX2 protein amount in the presence of Pi (Figure 2C,D). Likewise, bone morphogenic protein 2 (BMP2) was significantly increased by treatment with *miR‐134‐5p* mimic (Figure 2E). Osteoprotegerin (OPG) is a key regulator of bone formation^33^ and is also associated with vascular calcification.^34^ We observed that *miR‐134‐5p* mimic down‐regulated the expression of OPG (Figure 2F), suggesting that down‐regulation of a counter‐calcification signal may also participate in the *miR‐134‐5p*‐induced vascular calcification.

### 3.3 *miR‐134‐5p* targets HDAC5

**FIGURE 3:**
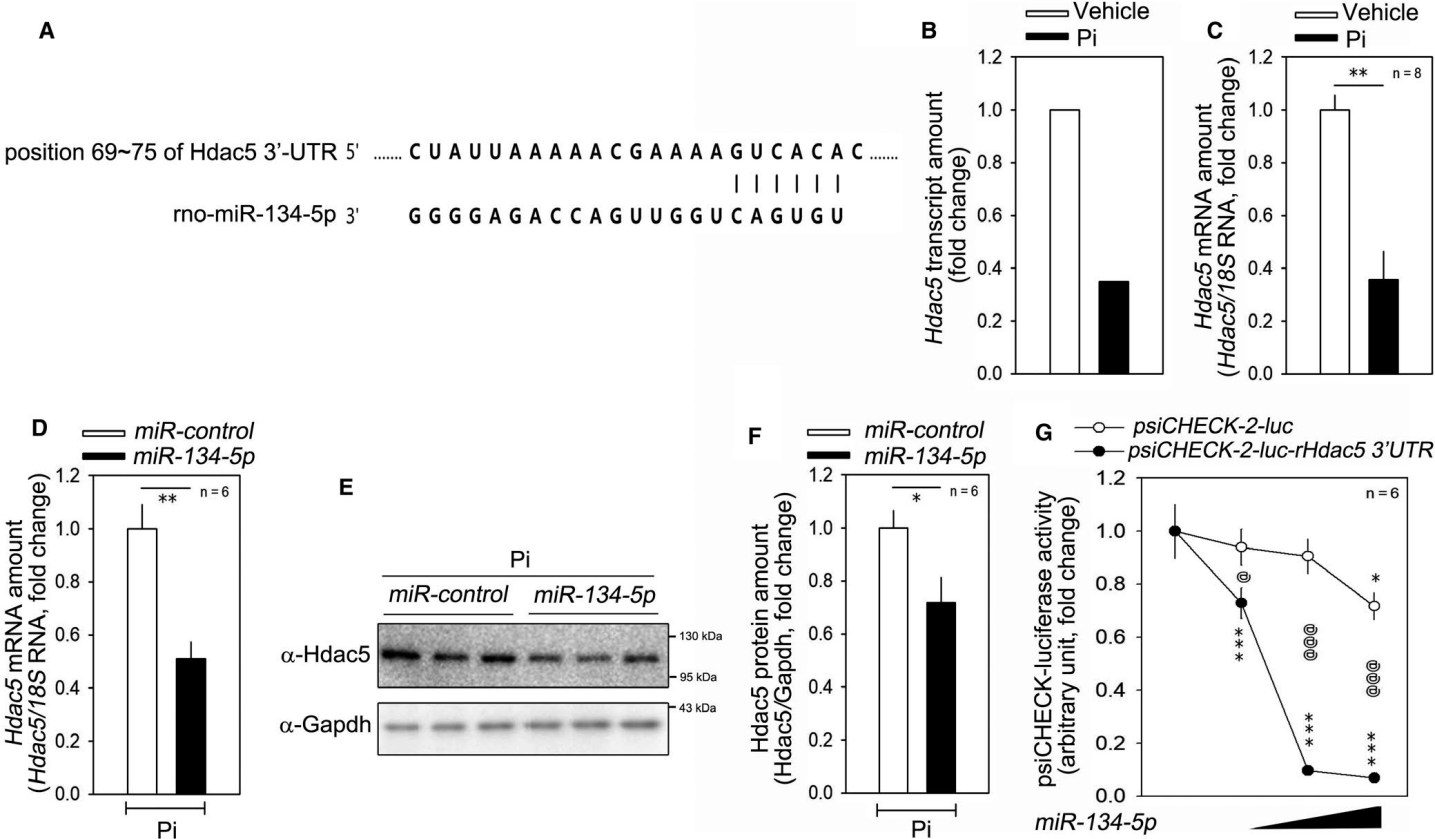
HDAC5 is a post‐transcriptional target of miR‐134‐5p. A, Sequence alignments of the HDAC5 3™UTR and miR‐134‐5p. B, mRNA microarray result showing that the HDAC5 mRNA amount was down‐regulated in Pi‐treated rat VSMCs. Two values were averaged. C, qRT‐PCR results. D, Transfection of miR‐134‐5p mimic reduced the mRNA level of HDAC5. qRT‐PCR results. E, miR‐134‐5p mimic reduced the protein amount of HDAC5 as determined by Western blot analysis. F, Quantification results are shown. G, miR‐134‐5p mimic significantly attenuated the luciferase activity driven by the HDAC5 3’UTR. Either psiCHECK™‐2‐luc rat HDAC5 3’UTR or psiCHECK™‐2‐luc empty vector was used to measure luciferase activity. * and @ *p* < 0.05. ** *p* < 0.01. *** and @@@ *p* < 0.001.

miRNAs induce degradation of their target mRNAs through direct binding to their 3′UTR, and the miRNAs and their target genes are reciprocally regulated.^4^ Thus, by in silico analysis as well as mRNA microarray, we searched for candidate targets of *miR‐134‐5p* that were down‐regulated in response to vascular calcification. Among the candidates, the 3′UTR of HDAC5 had a complementary sequence to *miR‐134‐5p* as shown in Figure 3A. mRNA array also showed that HDAC5 was down‐regulated by Pi treatment (Figure 3B). The Pi‐induced reduction in the HDAC5 transcript amount was further confirmed by qRT‐PCR (Figure 3C).

We observed that *miR‐134‐5p* could directly reduce the HDAC5 mRNA level (Figure 3D) and its protein level (Figure 3E). The quantitative results for the changes in protein level are shown in Figure 3F. Next, we generated a luciferase construct by inserting the 3′UTR of rat *HDAC5* into psiCHECK™‐2 vector. *miR‐134‐5p* mimic was transfected together with either psiCHECK™‐2*‐rHDAC5* 3′UTR plasmid or empty psiCHECK™‐2 vector. The *miR‐134‐5p* mimic successfully attenuated luciferase activity of psiCHECK™‐2‐luc*‐rHDAC5* 3′UTR, in a dose‐dependent manner, whereas it failed to do so with the empty vector (Figure 3G).

### 3.4 HDAC5 inhibits Pi‐induced vascular calcification

**FIGURE 4:**
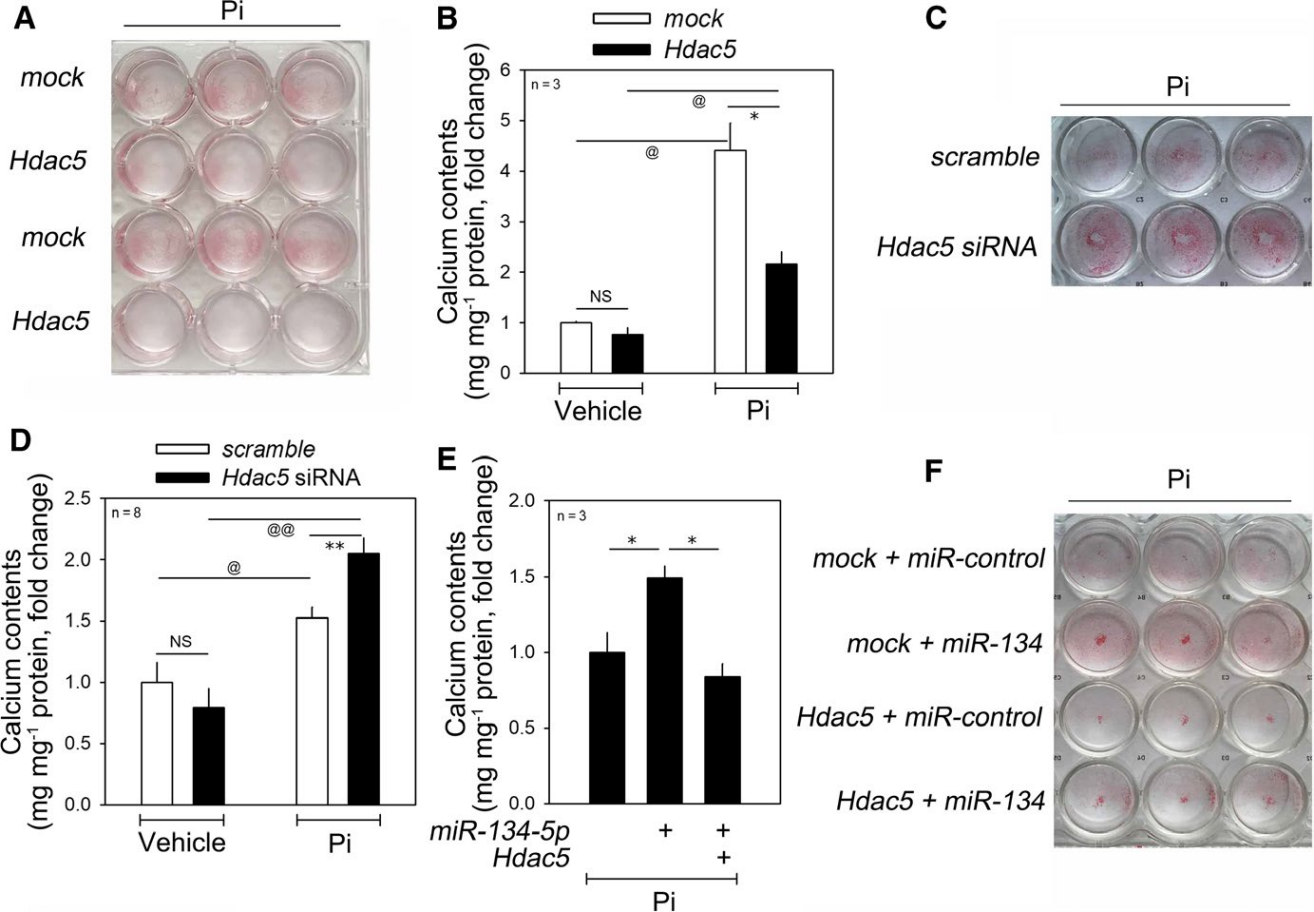
HDAC5, a novel anti‐calcification factor, mitigates *miR‐134‐5p*‐induced calcium deposition in the presence of Pi. A, Alizarin red S staining assay. B, Transfection of HDAC5 attenuated the increase in calcium deposition induced by treatment with Pi for 4 d. C, Alizarin red S staining. D, Knocking down of HDAC5 by *HDAC5* siRNA significantly enhanced the increase in vascular calcification induced by Pi treatment for 4 d. E, *miR‐134‐5p*‐induced increase in calcium contents was abrogated by simultaneous overexpression of HDAC5. F, Alizarin red S staining. * and ^@^ *P* < .05, ^@@^ *P* < .01. NS, not significant

HDAC5 was previously reported to inhibit RUNX2 induced by EGFR^35^ and TGF‐β.^36^ It has been also reported that *miR‐2861* inhibits HDAC5, thereby inhibiting the calcification of VSMCs.^14^ We therefore looked into the effect of HDAC5 on calcium deposition in VSMCs. In our experimental model, HDAC5 reduced Pi‐induced calcium deposition in VSMCs, as determined by Alizarin red S staining (Figure 4A). Transfection of HDAC5 did not significantly alter basal calcium contents in the absence of Pi (3 d, left two bars in Figure 4B). However, HDAC5 abolished Pi‐induced vascular calcification (right two bars in Figure 4B). By contrast, HDAC5‐knockdown by *HDAC5* siRNA potentiated the increase in calcium deposition induced by Pi treatment for 2 days (Figure 4C and right two bars in Figure 4D).

### 3.5 HDAC5 overexpression mitigates *miR‐134‐5p*‐mediated calcium deposition

To check whether *miR‐134‐5p*‐mediated vascular calcification is dependent on HDAC5, we tested whether transfection of HDAC5 could interfere with the effect of *miR‐134‐5p*. In the presence of Pi, transfection of *miR‐134‐5p* further enhanced the calcium deposition; the potentiation, however, was completely blunted by HDAC5 (Figure 4E). Alizarin red S staining further showed that *miR‐134‐5p*‐induced calcium deposition was attenuated by transfection of HDAC5 (Figure 4F).

## 4 DISCUSSION

**FIGURE 5:**
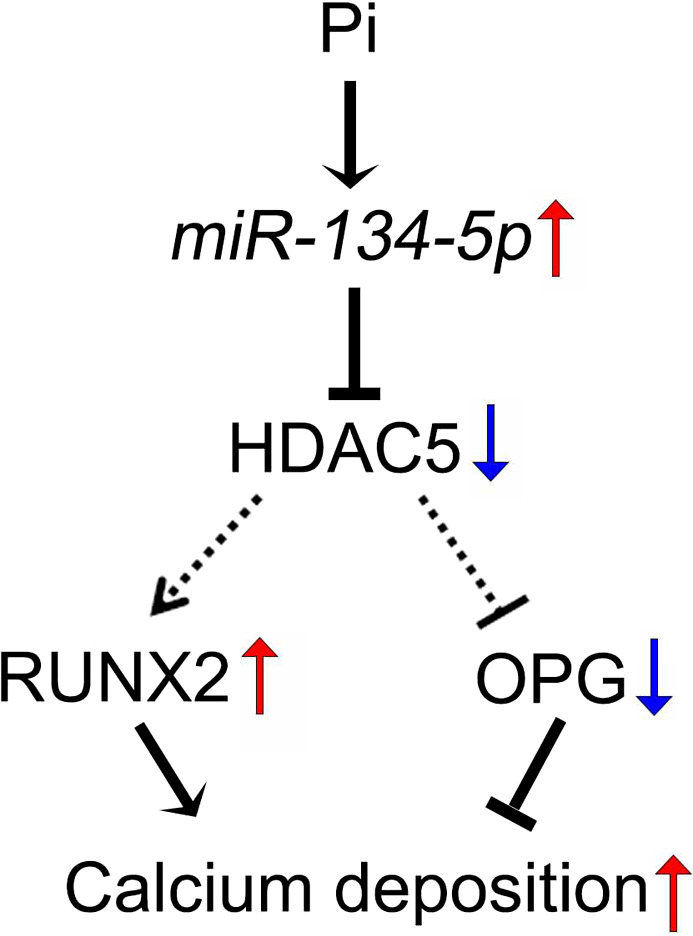
Diagram of *miR‐134‐5p/*HDAC5‐mediated calcium deposition in VSMCs. Treating VSMCs with Pi increased *miR‐134‐5p* transcription, thereby suppressing HDAC5 expression, followed by an increase in RUNX2 and decrease in OPG to induce vascular calcification

The present work illustrates a new mechanism of vascular calcification involving *miR‐134‐5p* and its target HDAC5. The main finding of this work is that stimulation of vascular calcification induces the expression of *miR‐134‐5p*, which in turn potentiates Pi‐induced calcium deposition by increasing RUNX2 and decreasing OPG (Figure 5). We also showed that *miR‐134‐5p* leads to the down‐regulation of HDAC5 and that HDAC5 works as an anti‐calcification mediator in VSMCs.

Given that they participate in the pathomechanisms of diverse diseases, noncoding RNAs are extensively involved in vascular calcification. For example, our research group previously reported that the long noncoding RNA *Lrrc75a‐as1* works as a negative regulator of vascular calcification.^37^ More recently, we also showed that circular RNAs such as *circSamd4a* reduce calcium deposition in VSMCs by absorbing miRNAs.^38^ In both of those previous studies, we noted that miRNAs are involved in the mechanisms of action of both the long noncoding and circular RNAs. Indeed, the most extensively investigated noncoding RNAs are miRNAs.^39^ Concerning the phenotypic effects of those RNAs, both positive^13^, ^14^ and negative^11^, ^12^ effects on vascular calcification have been reported.

Numerous studies have proposed a function for miRNAs in human diseases such as cardiovascular diseases. In addition, many miRNAs have been recognized as clinical, diagnostic and prognostic biomarkers, including in cancer. For example, *miR‐143/145* has been proposed as a marker of progression and metastasis of breast malignancy.^40^ Recently, several studies suggested *miR‐134* as a biomarker of cancer, because expression of *miR‐134* is markedly altered in many cancerous tissues.^41^, ^42^ It has been reported that *miR‐134* plays a key role in human carcinoma and participates in many aspects of cancer progression and has been associated with cancer prognosis in a variety of cancers.^41^ In addition, *miR‐134* directly regulates multiple target genes such as *DPD*,^43^ *FOXM1*,^44^ *KRAS* ^45^ and *STAT5B*,^46^ depending on type of carcinoma, through complicated signalling pathway including the MAPK/ERK signalling pathway, the EGFR pathways and the Notch pathway.^41^ However, the effects of *miR‐134* in cell proliferation and cancer progression are in debate, depending on the tumour type and target genes, whether tumour suppressor^47^, ^48^ or inducer.^49^, ^50^ It is noteworthy that *miR‐134* was first described to have a role in hippocampal neurons by regulating synaptic plasticity and memory.^51^ Recently, target genes of *miR‐134* such as *CREB*,^52^ *LIMK1* ^53^ and *PUM2* ^54^ have been identified in several studies.

The function of *miR‐134* has also been investigated in cardiovascular diseases, although fewer studies have been reported than for the nervous system or cancer. In the embryo, *miR‐134* regulates endothelium‐linked cardiac tube formation through down‐regulation of polycomb complex protein BMI‐1.^28^ Wu et al reported that *miR‐134* directly targets myeloid ecotropic insertion site 2 and promotes human cardiomyocyte precursor cell proliferation.^55^ Indeed, *miR‐134* might be induced in circumstances where active cardiomyocyte proliferation is required, since it is up‐regulated in acute myocardial infarction and could be an early marker of the disease.^56^ The function of *miR‐134* in myocardial infarction is to accelerate myocardial hypoxia/reoxygenation injury by targeting nitric oxide synthase 3, encoding endothelial nitric oxide synthase, which generates nitric oxide and plays a protective role in the cardiovascular system.^57^ Xiao et al revealed that inhibition of *miR‐134* could protect myocardial ischaemia/reperfusion injury through up‐regulation of NOS3 and activation of the PI3K/AKT pathway.^57^

*miR‐134* seems to actively participate in the progression of atherosclerosis not only in cardiomyocytes but also by targeting angiopoietin‐like 4 in macrophages.^58^ It is noteworthy that *miR‐134‐5p* is up‐regulated in the serum of patients with coronary artery calcification and could be used as a biomarker.^26^ However, to our knowledge, no research to investigate the mechanism of *miR‐134* in vascular calcification has been done. In the only report showing the function of *miR‐134* in VSMCs in the development of transverse aortic dissection,^29^ Wang et al found that *miR‐134‐5p* is down‐regulated in thoracic aortic dissection. They also observed that *miR‐134‐5p* induces VSMC differentiation by inducing the switch to the contractile phenotype and that STAT5B/ITGB1 is post‐transcriptionally down‐regulated.^29^ In the present study, we clearly demonstrated that *miR‐134‐5p* can potentiate Pi‐induced vascular calcification, although it alone did not induce calcium deposition. Indeed, *miR‐134‐5p* is a novel positive regulator of vascular calcification and remodelling.

HDAC5 is known to reduce RUNX2 activity in osteoblasts and miRNAs such as *miR‐2861* modulate osteoblast differentiation by targeting HDAC5.^18^ Since RUNX2 is a key player in both vascular calcification and bone formation, it is plausible that HDAC5 negatively regulates RUNX2 and thereby inhibits vascular calcification. Direct evidence between HDAC5 and vascular calcification, however, has not been fully demonstrated, whereas the involvement of HDAC4, an alternative class II HDAC, in vascular calcification has been reported.^59^ Rather, HDAC5 in association with HDAC4 is involved in VSMC hypertrophy and atherogenesis.^60^ One report showed that *miR‐2861* induces vascular calcification by targeting HDAC5.^14^ In the current study, we focused on down‐regulation of HDAC5 by *miR‐134‐5p* in bone‐like VSMCs, which resulted in vascular calcification. Although the binding of *miR‐134‐5p* to the HDAC5 3′UTR was sequence‐specific, this does not rule out the possibility that *miR‐134‐5p* reduces luciferase activity by binding to other, non‐predicted sites in the HDAC5 3′UTR. Therefore, a luciferase construct with modified or deleted target sequences of *miR‐134‐5p* may further confirm the specificity of the inhibition if the modification recovers luciferase activity. However, another remaining question is how HDAC5 is associated with vascular calcification, that is, whether suppression of HDAC5 by *miR‐134‐5p* directly or indirectly regulates the expression of osteogenic genes including RUNX2 and OPG in VSMCs, as well whether inhibition of *miR‐134‐5p* targeting HDAC5 effectively prevents vascular calcification in vivo. Thus, further studies are required to understand the precise mechanism by which the *miR‐134‐5p*/HDAC5 axis participates in vascular calcification. Those mechanisms could provide novel therapeutic approaches to treat diseases related to vascular calcification.

## CONFLICT OF INTEREST

The authors confirm that there are no conflicts of interest.

## AUTHOR CONTRIBUTION

**Nakwon Choe:** Data curation (equal); Investigation (lead); Writing‐original draft (equal); Writing‐review & editing (supporting). **Sera Shin:** Investigation (supporting). **Hosouk Joung:** Investigation (supporting). **Juhee Ryu:** Investigation (supporting). **Young‐Kook Kim:** Conceptualization (supporting); Data curation (supporting); Writing‐original draft (supporting); Writing‐review & editing (supporting). **Youngkeun Ahn:** Conceptualization (supporting); Data curation (supporting). **Hyun Kook:** Conceptualization (equal); Data curation (equal); Funding acquisition (equal); Supervision (equal); Visualization (lead); Writing‐original draft (equal); Writing‐review & editing (equal). **Duk‐Hwa Kwon:** Conceptualization (equal); Data curation (equal); Funding acquisition (equal); Supervision (equal); Visualization (equal); Writing‐review & editing (lead).

## Data Availability

The data that support the findings of this study are openly available in Gene Expression Omnibus (GEO) database under accession code GSE74755.
